# Beyond Trial Registration: A Global Trial Bank for Clinical Trial Reporting

**DOI:** 10.1371/journal.pmed.0020365

**Published:** 2005-10-18

**Authors:** Ida Sim, Don E Detmer

## Abstract

The prose reporting of trial information could be powerfully augmented by a computable repository of trial information-a global "trial bank."

## Background

A clinical trial is a research study in which human volunteers are treated and observed to answer a particular biomedical question. Clinical trials are one of the most valuable sources of evidence to determine which therapies are safe and effective. However, instances of selective reporting of results to benefit proprietary interests rather than public health have recently come to light. For example, in 2004, GlaxoSmithKline settled a US$2.5 million lawsuit for suppressing trial results showing that its antidepressant paroxetine (Paxil) increased suicidal ideation in children [1]. More recently, Merck and Pfizer have been criticized for with-holding results showing increased risk of heart disease from COX-2 drugs such as rofecoxib (Vioxx) [2–4], which was withdrawn from the market because of these risks.

A complete public register of trials and the subsequent release of all results are crucially important to prevent drug and device makers from skewing the public record on the effectiveness of therapies. However, even when local laws require that trials be registered, compliance has been incomplete. In the United States, the Food and Drug Administration Modernization Act [5] requires that all trials on life-threatening diseases be registered into ClinicalTrials.gov (a register maintained by the National Institutes of Health), yet only 48% of industry-sponsored trials were registered during the initial period of the law's implementation [6]. Moreover, trials are sometimes registered with uninformative data (e.g., not giving the name of the tested drug) [7], thus subverting the central purpose of registration, which is to increase transparency.

In reaction to this general state of affairs, an influential group of medical journal editors recently declared that they will publish only previously registered trials [7,8]. In addition, legislation is being introduced in many jurisdictions to impose broader mandates on trial registration and reporting (e.g., Fair Access to Clinical Trials Act in the US Congress). The World Health Organization (WHO), recognizing the highly international nature of modern clinical trial conduct, is establishing policies and standards for trial registration and reporting worldwide [9]. A global commitment is, thus, emerging to ensure that key information about all clinical trials are registered, and that each trial's results are fully reported.

At a time when over 20,000 new trials are initiated worldwide each year, and with over 294,000 trials already indexed in PubMed, careful thought should be given to how computers could be used to manage the deluge of information. The current consensus is to attempt to code some of the registration data fields (e.g., condition, interventions, and outcomes) using a standard medical vocabulary (e.g., International Classification of Diseases [10] and SNOMED [11]), and to report trial results in English in at least a PDF version of the International Conference on Harmonisation E3 guidelines (http://www.ich.org/). However, computers cannot read or understand prose very well at all. For example, the sentence “mean creatinine was 1.9 (95% confidence interval, 1.2–2.6) in the intervention group and 2.4 (95% confidence interval, 1.9–2.9) in the comparison group” is not directly usable by search engines, statistical programs, or decision support systems. The prose reporting of trial information could be powerfully augmented by a computable repository of trial information—a global trial bank. Unlike prose, computable information is structured and coded for computation and allows the use of advanced information technologies for knowledge management. With billions of dollars spent annually on drugs and other health interventions, the world cannot afford to keep knowledge from clinical trials only in prose. [Fig pmed-0020365-g002]

**Figure pmed-0020365-g002:**
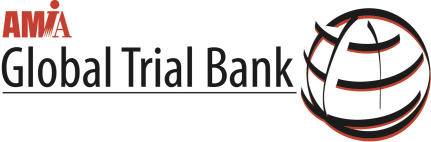
Global Trial Bank logo

## The Global Trial Bank Project

Global Trial Bank (GTB) is a nonprofit organization formed under the auspices of the American Medical Informatics Association (http://www.amia.org), a professional scientific association. GTB's goal is to speed the dissemi-nation, understanding, synthesis, and translation of clinical trials to improve human health. To reach this goal, GTB seeks to make available open-access and computable peer-reviewed results from all clinical trials conducted worldwide, regardless of whether the results are favorable or not, to provide an unbiased knowledge base for improving human health. Through the GTB Web site (http://www.globaltrialbank.org), clinicians and patients will be able to accurately retrieve relevant trial results, and scientists will be able to analyze data across trials to compare and contrast trials and to generate new findings and insights. The key features of GTB are as follows. [Fig pmed-0020365-g001]

**Figure pmed-0020365-g001:**
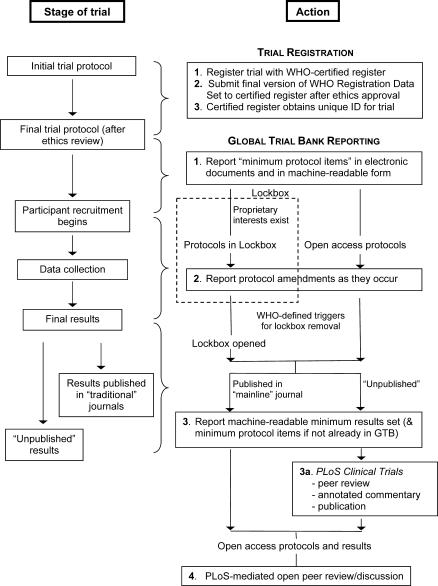
Schematic showing relationship between trial registration, Global Trial Bank reporting, and *PLoS Clinical Trials* (Image: Ida Sim)

### Sufficient detail for science

GTB will collect necessary protocol and results information in sufficient detail to allow the scientific community to assess a study's scientific strengths and weaknesses, and to properly interpret the findings. This includes information on study design (e.g., allocation concealment), study execution (e.g., withdrawal rates and compliance), and study results (e.g., subgroup results and adverse events). The data collected will be compatible with the WHO Registration Data Set [9] as well as the CONSORT statement on trial reporting (http://www.consort-statement.org/).

### Computable information

GTB will collect the following information in structured, coded form: eligibility criteria, interventions, outcomes, and summary results for outcomes at all predefined time points for all intervention and comparison groups and predefined subgroups. GTB will then be able to support queries such as “retrieve all trials on women over age 60 with heart disease in which a beta-blocker was studied, and that report heart attack rates at three and five years for which at least 80% of the participants were followed up.” Such detailed queries and analyses will enable much more powerful com-puter-assisted interpretation, application, and data mining of clinical trial information than is possible today.

To further support clinical trial knowledge management, GTB will provide standards-compliant interfaces for information and decision support systems to directly access the GTB database over the Internet. This will allow third parties to provide customized solutions without having to manually transcribe data from text articles to another computer.

### Integrated peer review

We anticipate that protocol information will be either downloaded directly from a trial register if not registered directly with GTB or entered by the trialist. Protocol information will not be peer reviewed. It is critical, however, that trial results be peer reviewed. Results will come from three sources: (1) results reported in peer-reviewed mainline journals, (2) results reported in non-peer-reviewed publications or Web sites (e.g., pharmaceutical company sites), and (3) results reported directly to GTB.

Results not directly reported to GTB will be captured semiautomatically with some input by GTB staff and/or the trialist. GTB will link to peer-reviewed publications and will not perform further peer review on these trials. There is a precedence, called “trial bank publishing” [12], for publishing trial results as both prose journal articles and trial bank entries. It is a model akin to the publication of genomic information in both GenBank and traditional scientific journals [13].

Trial results that have not been previously peer reviewed will be submitted to *PLoS Clinical Trials* (http://www.plosclinicaltrials.org), a new PLoS journal, for peer review. *PLoS Clinical Trials* anticipates accepting all trials that meet a set of minimum require-ments regardless of outcome or “clinical interest” to maximize the number of trials that are reported to the public. PLoS will also provide annotated commentaries on accepted studies and a discussion forum for open postpublication peer review of all GTB entries of completed trials.

### Promotes transparency, interoperation, and open access

GTB fully supports international efforts to standardize and streamline trial registration and reporting. To this end, all GTB trials must be registered with a WHO-certified trial register using the WHO global unique ID (see http://www.who.int/ictrp), and all GTB data exchange interfaces will be compatible with relevant emerging stan-dards (e.g., Clinical Data Interchange Standards Consortium, Health Level Seven, WHO, and Cancer Biomedical Informatics Grid). In addition, GTB strongly supports open access to clinical trial information: patients, clinicians, and scientists will be able to search, browse, analyze, and download GTB data without charge or restriction.

GTB builds upon the National Institutes of Health–funded research of the Trial Bank Project (http://rctbank.ucsf.edu/) and PLoS's vision of a new, more inclusive approach to make peer-reviewed clinical trial results available to the public. GTB has formed an international advisory board and received a seed grant, and is actively forming additional partnerships and seeking foundation funds for start-up.

## Challenges

GTB reflects a new approach to publishing clinical research: publishing in both prose and computable form, as well as publishing all results, not just “clinically important” ones. As such, GTB faces undeniable challenges. The work of data entry must be reasonable. The coding of data fields in a controlled medical vocabulary (e.g., SNOMED) must be relatively easy and reproducible. The publishing model should result in overall strengthening of the quality and usefulness of clinical trial publishing. And finally, the operation must be open access yet financially self-sustaining. Intermediate solutions to these challenges are available and will provide opportunities for continuing improvement.

## Conclusions

The reporting of clinical trial results as both prose and computable data is arguably a natural progression in the development of electronic publishing. Coupled with emerging international policies on clinical trial registration, GTB offers the most advanced computable repository of trial protocol and results information to promote biomedical discovery as well as transparency and accountability in clinical trial research. With its additional features of being nonprofit, open access, and peer reviewed, we anticipate that GTB will become a major global resource for knowledge management in biomedicine.

## Abbreviations

GTB: Global Trial Bank
WHO: World Health Organization

## References

[pmed-0020365-b1] New York Supreme Court (2004). People of the State of New York v. GlaxoSmithKline.

[pmed-0020365-b2] Topol EJ (2004). Failing the public health—Rofecoxib, Merck, and the FDA. N Engl J Med.

[pmed-0020365-b3] Mathews A, Martinez B (2004 November 1). E-mails suggest Merck knew Vioxx's dangers at early stage. Wall Street Journal.

[pmed-0020365-b4] Psaty BM, Furberg CD (2005). COX-2 inhibitors—Lessons in drug safety. N Engl J Med.

[pmed-0020365-b5] United States Congress (1997). The Food and Drug Administration Modernization Act of 1997, Pub. L. No. 105–115, 113. http://www.fda.gov/cder/guidance/s830enr.txt.

[pmed-0020365-b6] Derbis J, Toigo T, Woods J, Evelyn B, Banks D (2003). FDAMA section 113: Information program on clinical trials for serious and life-threatening diseases [abstract].

[pmed-0020365-b7] De Angelis CD, Drazen JM, Frizelle FA, Haug C, Hoey J (2005). Is this clinical trial fully registered? A statement from the International Committee of Medical Journal Editors. Ann Intern Med.

[pmed-0020365-b8] DeAngelis CD, Drazen JM, Frizelle FA, Haug C, Hoey J (2004). Clinical trial registration: A statement from the International Committee of Medical Journal Editors. JAMA.

[pmed-0020365-b9] World Health Organization (2005). International clinical trials registry platform.

[pmed-0020365-b10] World Health Organization (2005). International classification of diseases (ICD).

[pmed-0020365-b11] SNOMED International (2005). SNOMED CT.

[pmed-0020365-b12] Sim I, Carini S, Olasov B, Jeng S, Fieschi M, Coiera E, Li YCJ (2004). Trial bank publishing: Phase I results. MedInfo 2004: Proceedings of the 11th World Congress on Medical Informatics.

[pmed-0020365-b13] Benson DA, Karsch-Mizrachi I, Lipman DJ, Ostell J, Wheeler DL (2005). GenBank. Nucleic Acids Res.

